# Characterization of plasma protein binding dissociation with online SPE-HPLC

**DOI:** 10.1038/srep14866

**Published:** 2015-10-13

**Authors:** Ping Li, Yiran Fan, Yunlong Wang, Yaxin Lu, Zheng Yin

**Affiliations:** 1 College of Pharmacy & State Key Laboratory of Elemento-Organic Chemistry, Nankai University, Tianjin 300071, P.R.China; 2 Collaborative Innovation Center of Chemical Science and Engineering (Tianjin), Tianjin 300071, P.R.China; 3 Tianjin International Joint Academy of Biomedicine, Tianjin 300457, P.R.China; 4 School of Mathematical Sciences, Nankai University, Tianjin 300071, P.R.China

## Abstract

A novel parameter of relative recovery (R_re_) was defined and determined by online SPE-HPLC to characterize plasma protein binding (PPB) kinetics of highly plasma binding drugs. The proportional relationship of R_re_ with k_off_ of PPB has been established with a new SPE model. A rapid, easy to use method could potentially be used to categorize PK properties of the drug candidates in the decision process of drug discovery and development.

The binding of a drug to proteins and lipids in plasma (termed plasma protein binding (PPB)) is an unavoidable process after a drug being distributed in circulating blood. Free drug theory (FDT)^1^,^2^,^3^ is widely accepted to explain how the PPB of a drug relates to its *in vivo* phenomena: the binding of a drug is taken as a reversible and rapid equilibrium process^2^,^4^ resulting in the constant and instantaneous concentration of unbound (free) drug. And only the free drug can enter tissues, get to the sites of action/metabolism, and exert its effect for some useful period of time. Therefore PPB has profound effects on both the pharmacodynamics (PD) and pharmacokinetics (PK) profiles of a drug because the free drug concentrations surrounding the action and metabolic targets determine the overall behavior of a drug.

In the traditional study of drug-protein binding, the binding thermodynamics based on FDT is mainly studied^5^. Measurement of the bound drug fraction of a compound (percentage of plasma protein binding) using an *in vitro* PPB assay is a common practice in drug discovery and is a requirement for new drug application. Based on the assumption of drug-protein instantaneous equilibrium, many approaches have been developed to assess PPB, for example equilibrium dialysis (ED), ultrafiltration (UF), ultracentrifugation (UC), LC techniques, capillary electrophoresis (CE), spectroscopy and calorimetric techniques, etc^4^,^6^. The percentage of plasma protein binding is a value related to the equilibrium constant in a permanent state. The data is often used not only to rationalize *in vivo* phenomena of a drug but also to guide the medicinal chemistry efforts in lead optimization and candidate selection process.

However, the practice of percentage of plasma protein binding neglects the great influence of the binding kinetics in dynamic living organisms^7^. The drug-protein binding can be described by the following equilibrium in the simplest case^4^,^8^:


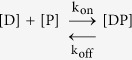


where [D], [P], and [DP] are the unbound drug, unbound proteins, and drug-protein complex concentrations respectively, and k_on_ and k_off_ are the association and dissociation rate constants. Thus the dissociation constant (K_d_) can be defined as follows^4^,^9^:


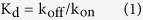


The binding kinetics can be described^10^,^11^:


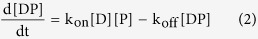


The assumption of drug-protein instantaneous equilibrium is valid in most *in vivo* study because the time scale of protein-binding equilibration is much shorter than that of other biological processes, for example, drug distribution and clearance. But this may be an incorrect assumption when the dissociation of unbound drugs from the plasma protein may become one of the limiting steps under several circumstances^1^,^2^,^3^, for instance: drug-protein dissociation rate is slower than drug clearance from plasma. In such cases, the kinetics of drug-protein binding could act as a major determinant^7^ and should be equally valued in the process of rationalizing *in vivo* phenomena because the dissociation of drug from plasma could become rate limiting step. The influence of PPB kinetics complicates the understanding of *in vivo* phenomena of a drug. If a drug molecule is highly (low percentage of unbound drug) and tightly (slow dissociation) bound to plasma protein, the possible effects of the PPB could be: (a) the drug is kept in the plasma chamber; (b) drug distribution is limited to the target tissue (reduced volume of distribution); (c) observed metabolism and clearance is reduced (extended half-life); (d) brain permeability is limited; (e) a higher starting dose and a lower maintenance dose are required. Various approaches have been reported in the literature to study the kinetics of PPB including stopped-flow spectroscopy^12^, fluorescence quenching^6^, chromatography frontal analysis^7^,^12^, and surface plasmon resonance (SPR)^6^. However, these approaches have seen only very limited use for studying the rate of drug interactions with only specific component of plasma, such as HSA, instead of whole plasma^12^. In addition, they normally involve rather complicated process. We sought to devise a quick, simple, practical method to characterize PPB kinetics of a drug with whole plasma and thereby explain and predict its *in vivo* behaviors.

Solid phase extraction (SPE)^13^,^14^ is a sample preparation process developed to enrich and isolate the analytes of interest from matrix. Recently, the fully automated online SPE coupled with high performance liquid chromatography (HPLC) has been established for the quantitative determination of drug concentration in plasma for pharmacokinetic study^15^. The online SPE system to isolate and enrich a drug from plasma is a dynamic system, which involves the association-dissociation process of drug-plasma protein binding, and adsorption and desorption process between unbound drug and SPE column (Fig. 1). K_ad_ was the adsorption constant of the SPE cartridge to unbound drugs. Hypothetically, the SPE adsorption of a drug could be a reflection of PPB kinetics through the competition of free drugs with PPB in the dynamic elution process. We speculated that relative recovery rate (R_re_) of SPE, which represents the retention capability of SPE cartridge to a drug in presence of plasma, could be correlated to the dissociation rate constant of PPB. Herein a novel parameter of R_re_ was defined and determined using online SPE-HPLC. The relationship study of R_re_ with PPB dissociation rate constant was studied with a SPE model. And most importantly, R_re_ was used to categorize PK properties of drugs. This study provides a novel, simple and easy to use approach to characterize PPB dissociation of a drug and thereby help to explain and predict the *in vivo* phenomena of a drug.

Pharmacokinetics (PK) is the study of processes impacting drug exposure in an organism in particular absorption, distribution, metabolism and elimination (ADME). It is a standard practice for drug development. PPB has significant influence on PK properties of a drug (Supplementary Table 1). Generally, PPB may influence observed drug clearance (CL) and volume of distribution (V_d_) through its impact on the diffusion rate of drugs between blood plasma and tissue^1^. PPB has a rather profound effect on both the CL and the V_d_, thereby the half-time (T_1/2_) of a drug that is a product of the CL and the V_d_. Both thermodynamics and kinetics properties of PPB may have influence on PK. The thermodynamics of PPB (percentage of plasma protein binding) has been well studied and widely applied in pharmaceutical research to explain *in vivo* behavior of a drug because the instantaneous equilibrium could be reached in most cases and kinetics has little impact. However, PPB kinetics may play significant roles in PK of drugs with high protein binding and slow dissociation rate since the dissociation of unbound drugs from the plasma protein may become the rate limiting step. If a drug has high percentage binding to plasma protein and slow dissociation from plasma protein, the effect of the binding on the ADME of the drug could be ‘restrictive’ to drug retention in plasma. By contrast, if a drug has high percentage binding and fast dissociation or low percentage binding and slow dissociation, or low percentage binding and fast dissociation, the effect of the plasma protein binding on ADME can be ‘nonrestrictive’ or ‘permissive’^16^. In the case of drugs with high percentage binding to plasma protein, the differentiation of “restrictive” and “nonrestrictive” effect is determined by dissociation kinetics of PPB. Therefore, drugs with high percentage binding to plasma protein could well serve as a good model for the study of impact of PPB kinetics on PK. In addition, a high quality unified PK dataset of drugs in the public domain provides the opportunity to perform such model study. In order to study the impact of PPB kinetics on PK, a total of 13 drugs characterized as the percentage of protein binding greater than 90%^16^,^17^ were selected for this study (Supplementary Fig. 1). They could be further divided into two groups: drugs with short half-life (T_1/2_ < 4 h), and drugs with long half-life (T_1/2_ > 24 h)^18^ (Table 1). This research was envisaged to develop a SPE-HPLC approach that could characterize the PPB kinetics with a novel parameter of R_re_. Consequently, the influence of PPB kinetics on PK properties of drugs rather than PPB thermodynamics has been studied, and the relationship of PPB kinetics parameters (k_off_) and PK properties (T_1/2_) has been explored.

## Results

The retention time of the conventional HPLC system (T_1_), the retention time of the online SPE system (T_2_), the absolute recovery (R_ab_) and the relative recovery (R_re_) of 13 analytes were shown (Table 2). Even though all 13 drugs have high percentage of plasma protein binding, the relative recoveries were very different, ranging from 21.6 to 107.5%, while their absolute recoveries were high (91.8 ~ 102.6%). Dicoumarol, phenylbutazon, piroxicam, warfarin sodium and estriol had low R_re_. Low R_re_ means only a small part of unbound drugs were retained in SPE column when the drugs were loaded on SPE column in presence of plasma. Hypothetically, the slow dissociation rate of drug-PPB complex leads to drug being washed out with PPB by mobile phase before complete dissociation of drug-PPB complex. In the case of high R_re_, when the dissociation rate of drug-PPB is fast, a rapid equilibrium is achieved resulting in drug being retained in SPE cartridge while plasma protein washed out.

**Figure 2:**
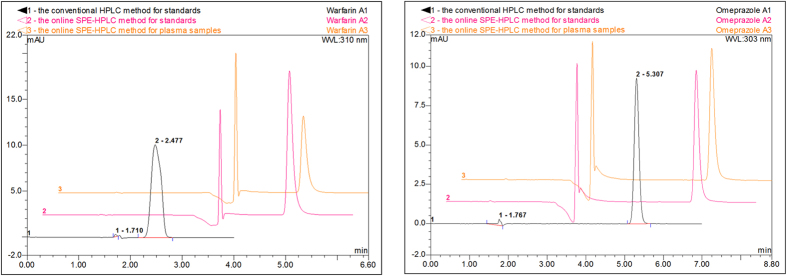
The comparison charts of warfarin sodium (left) and omeprazole (right) (1-interference peak, 2-drugs peak).

In example warfarin sodium (low R_re_) and omeprazole (high R_re_), the comparison charts of their peak area were shown (Fig. 2).

## Discussion

**Figure 3:**
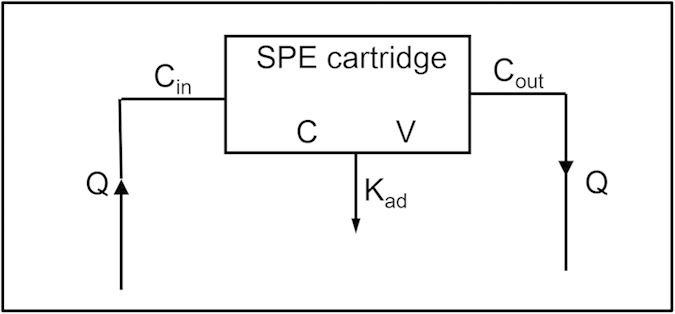
The online SPE model of drugs. Q represented the flow rate of SPE cartridge, C_in_ and C_out_ represented respectively the drug concentration in the incoming and outgoing SPE cartridge. C and V were separately the drug concentration in SPE cartridge and the volume of SPE cartridge. K_ad_ represented the adsorption constant of the SPE cartridge to drugs.

Experimentally, drugs with similar PPB thermodynamic properties (percentage of plasma protein binding greater than 90%) had significantly different R_re_. Hypothetically, R_re_ of drugs could reflect the kinetics of PPB and could be related to dissociation rate of drug-PPB complex. In order to verify this hypothesis, a SPE model was established to rationalize the experimental data (Fig. 3).

### A new SPE model - the proportional relationship of R_re_ with k_off_ of PPB

Similar to the simplified hepatic clearance model^11^,^19^, the assumption of the SPE model was that a SPE system was a single well-stirred compartment and the unbound drug was rapidly in equilibrium between plasma and SPE column. Experimentally, plasma protein was flushed out of the SPE column on 0.1 min after sample injection. Through the SPE experiment, plasma protein concentration is far greater than the concentration of drugs and the dilution effect could well be neglected. The influence of the PPB kinetics in the plasma was investigated on the SPE system.

The SPE model was established as following:

(i) Based on FDT, for a linear system, the unbound protein concentration can be assumed constant and equal to the total protein concentration in plasma, P_o_^11^. The unbound drug fraction, f_u_, in plasma is then calculated as:


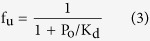


Replacing K_d_ in equation (1), according to equation (3) as:


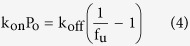


(ii) The rate of change of drug quantity, dA/dt, inside SPE cartridge was provided by the drug entering and exiting the SPE column, dA_∆_/dt, and by drug adsorption inside the SPE column, dA_ad_/dt, so that


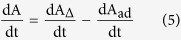


The rate of drug quantity change inside the SPE cartridge due to the mobile phase flow was calculated as


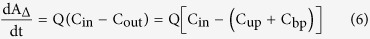


where C_in_ and C_out_ were separately the drug concentration in the incoming and outgoing mobile phase. The concentration of drug inside SPE system was provided by the unbound, C_up_, and bound, C_bp_, drug concentrations in plasma. Q was the flow rate of SPE cartridge.

The rate of drug adsorption was calculated as


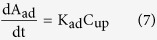


where K_ad_ was the adsorption constant of the SPE cartridge to drugs.

Substituting equation (6) and (7) in equation (5) finally yielded the equation for the quantity of drug inside SPE cartridge, so


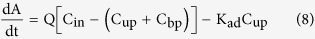


(iii) The rate of change of bound DP concentration inside SPE cartridge, dC_bp_/dt, was provided by the kinetics of drug–protein binding, d[DP]/dt (equation (1)), and by the bound drug entering and exiting the SPE cartridge, dC_∆_/dt, so that,


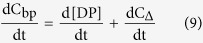



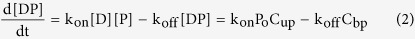


Similar to the above equation (6), the rate of bound drug quantity change, dA_bp,∆_/dt, inside the SPE cartridge due to the mobile phase flow was calculated as


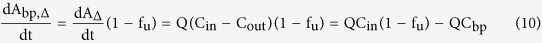


The rate of bound drug concentration change inside the SPE cartridge due to the mobile phase flow was calculated as


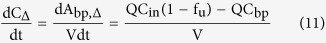


where V was the volume of SPE cartridge.

Substituting equation (2) and (11) in equation (9) finally yielded the equation for the bound drug concentration inside SPE cartridge, so


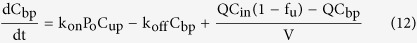


(iv) A steady state was assumed to exist in the online SPE system, we could set the steady-state condition for equation (8) and (12),


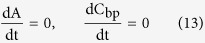


So we would get the system of two linear equations for 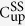 and 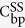.


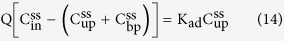



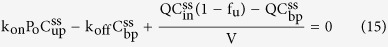


where 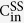 was the steady-state drug concentration in the incoming mobile phase. 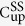, 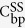 were respectively the steady-state unbound and bound drug concentrations in SPE column. This would allow expressing 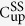 and 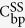 through 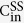.

(v) We defined E_ss_ as the steady-state rate of drug adsorption into SPE cartridge, according to equation (5) and (6), we got


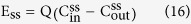


Using equation (6) and (14), we obtained the equation for E_ss_,


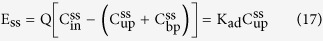


For online SPE system, we could define relative recovery R_re_ through E_ss_


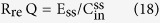


Finally, we could solve R_re_ according to equation (14), (15), (17) and (4), the equation for R_re_ could be arranged as


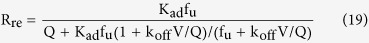


Based equation (19), the k_off_ could be estimated directly through determination of R_re_ of the drugs in experiment. Four drugs such as phenylbutazon, warfarin sodium, nimodipine and omeprazole were chosen to build the SPE model.

**Figure 4:**
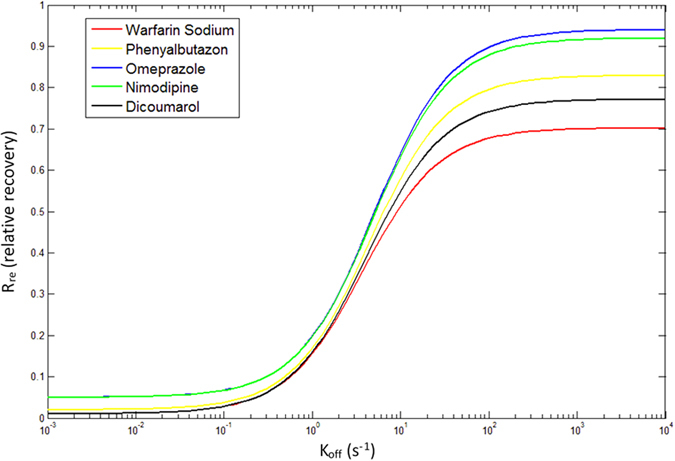
The relationship of R_re_ and k_off_ of the drugs.

Using Eq. 19, the relationship of R_re_ and k_off_ of four drugs could be solved (Fig. 4). Calculations were performed for the online SPE system, Q = 1 mL/min, V = 0.1884 mL, K_ad_ = 700 logP^−1^ (K_ad_ was assumed 700 logP^−1^ in order to achieve the online SPE model, which was consist to practical significance of K_ad_ because drugs adsorption rate was related to logP). Finally, dicoumarol was chosen to verify this model (Fig. 4).

As expected, the k_off_ values of dicoumarol, phenylbutazon and warfarin sodium were small but nimodipine and omeprazole were large tending to infinity. The k_off_ values of dicoumarol, phenylbutazon and warfarin sodium were of the order: dicoumarol < phenylbutazon < warfarin sodium, which indicated R_re_ value was proportional to the k_off_ value.

Experimentally, R_re_ of drugs with similar high percentage of plasma protein binding were significant different. In SPE model study, R_re_ value was proportional to the k_off_ value than percentage of plasma protein binding. The data implies that the drugs with low R_re_ has small k_off_ value, and the drugs with high R_re_ has large k_off_ value. The SPE model could verify well our hypothesis. Similarly, the k_off_ values of other drugs could also be solved by measuring R_re_ value.

To our knowledge, there are lack of calculated or measured k_off_ values of drugs with the whole plasma protein. In our study, k_off_ values could be solved based on SPE model. However, there were reasonable assumptions made to simplify the analysis. k_off_ solved by SPE model is rather an empirical value than absolute value. The best use of k_off_ values solved by SPE model could be the comparison of the relative strength of how tightly drugs bind to plasma protein.

### The application of R_re_ to correlate the k_off_ of PPB with half-time

PK properties are of great importance in lead optimization. Chemist needs to have a rapid and efficient way to quickly identify the right candidate. It has been a challenge for chemist to fully understand the impact of PPB on PK properties and thereby make the right decision in compound selection process. The plasma half-life is governed by volume of distribution and plasma clearance according to the equation: T_1/2_ = 0.693 × V_d_/CL^20^. PPB has a significant effect on the diffusion rate of drugs between blood plasma and tissues. The influence of PPB on the CL depends on drug intrinsic clearance and the V_d_ is related to the free fraction of drugs in plasma and tissues. Therefore PPB has rather profound impact on PK properties and it is of great challenge to predict the impact of PPB on the CL and the V_d_, thereby half-time of a drug^21^. At present, the percentage of plasma protein binding has been used widely for *in vivo* PK research of a drug, and the kinetic parameter has been tend to be neglected. However, under certain circumstances, kinetics of PPB may play a crucial role in the life-time of a drug. For instance, it has been suggested that drugs that have slow drug-PPB complex dissociation rate would have long half-time^22^,^23^. Since R_re_ was proportional to k_off_ and k_off_ and T_1/2_ was related, the relationship analysis between R_re_ and T_1/2_ was investigated. The PK parameters and R_re_ values of drugs were analyzed (Table 3). According to R_re_ and T_1/2_, all 13 drugs were characterized as three categories: the first class had long T_1/2_, low R_re_; the second had long T_1/2_, high R_re_; the third had short T_1/2_, high R_re_. It was clearly found that drugs with low R_re_ that is proportional to small k_off_ have long T_1/2_. The observation could be reasoned that the slow dissociation of drugs from plasma protein may slow down the diffusion of the drugs to the metabolic tissues, which may result in the long half-time.

## Conclusions

In summary, a novel parameter of relative recovery (R_re_) was defined and determined by online SPE-HPLC to characterize drug plasma protein binding kinetics. The proportional relationship of R_re_ with k_off_ of PPB has been established with a new SPE model. And importantly, a rapid, easy to use method could potentially be used to categorize PK properties of the drug candidates in the decision process of drug discovery and development: compounds with low R_re_ would have small k_off_ that lead to long T_1/2_.

## Methods

### Samples preparation

The stock solutions of amitriptyline hydrochloride, estradiol, estriol, fluoxetine hydrochloride, indomethacin, nimodipine, norfluoxetine hydrochloride, omeprazole, phenylbutazon and piroxicam were prepared in acetonitrile (Merck KGaA, Germany) respectively. Two stock solutions of warfarin sodium and diclofenac sodium were separately prepared in water. The stock solution of dicoumarol was prepared in DMSO. All stock solutions were stored at 4 °C. Working standard solutions were completely diluted to 100 μg/mL from these stock solutions with 10 mM phosphate buffered saline (PBS) (pH 7.4). Test standards were further diluted to 10 μg/mL with 10 mM PBS (pH 7.4) (10/90, v/v) and plasma samples were prepared to 10 μg/mL by adding 100 μg/mL standard solutions into blank human plasma (The Chinese people’s Liberation Army 301 Hospital, Beijing, China) (10/90, v/v). The final concentration of test standards and plasma samples were both 10 μg/mL.

### Online SPE-HPLC measurements

HPLC analyses was performed using a Dionex UltiMate 3000 × 2 Dual-Gradient HPLC system (Sunnyvale, CA, USA). The chromatographic separation was performed on an Acclaim^TM^ 120 C18 column (5 μm, 4.6 mm × 150 mm, Thermo Scientific). LiChrospher® RP-18 ADS(25 mm × 4 mm, 25 μm, Merck) (Darmstadt, Germany) and Oasis® MAX (2.1 mm × 20 mm, 30 μm, Waters)(Milford, MA, USA) were used as on-line extraction column. The injection volume was 5 μL. The temperature of sample plate and the column compartment was set at 37 °C. The mobile phase that consisted of acetonitrile-10 mM PBS (pH 7.4) conditions and UV spectra were listed (Table 4).

**Figure 5:**
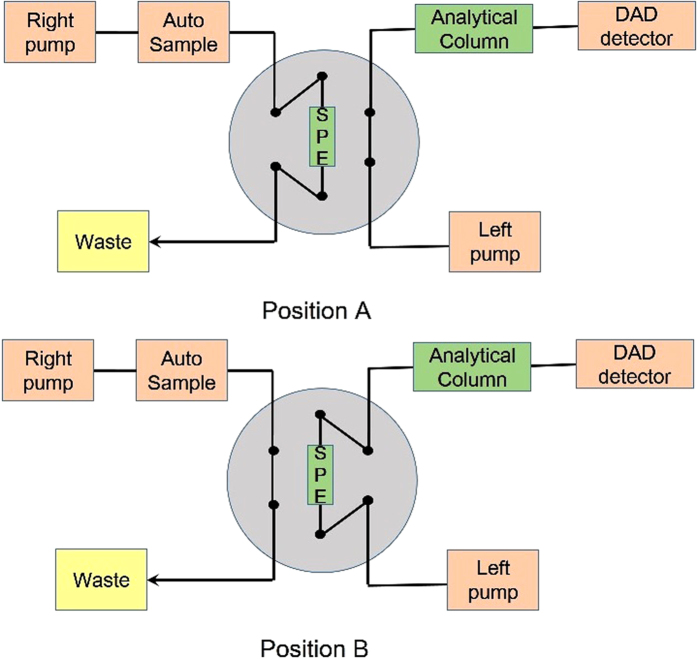
The schema for online SPE-HPLC setup.

The setup of the online SPE-HPLC method is shown (Fig. 5). Test standards or plasma samples were loaded onto a SPE cartridge. A Valco 6-port switch valve controlled by the HPLC system was used to direct the flow path. The online SPE cartridge switching strategy for extractive sample and analysis included four steps^24^ (Fig. 5). (a) Loading. The valve was at position A. The right pump was used to load 5 μL of test standards or plasma samples to SPE cartridge. The samples on the SPE cartridge were washed for 1 min with 100% PBS at a flow rate of 1 mL/min. Simultaneously, The left pump was used to balance the analytical column. At last the sample matrix was flushed to waste, the analytes were retained on the SPE column. (b) Reversed-phase elution. The valve was switched to position B that the SPE cartridge was couple with the analytical column. The samples were back eluted from the SPE cartridge onto the analytical column for 1 min by acetonitrile-10 mM PBS buffer (pH 7.4) (volume ratio was shown in Table 4) at a flow rate of 1 mL/min by the left pump. (c) Separation. After the transfer of the analytes onto the analytical column, the valve was switched back to position A. Separation of the analytes was achieved on the analytical column with acetonitrile-10 mM PBS buffer (pH 7.4) (volume ratio was shown in Table 4) at a flow rate of 1 mL/min. (d) Re-equilibration. During the analytical separation, the SPE column could be washed and re-equilibrated with 100% PBS at a flow rate of 1 mL/min.

### Online SPE-HPLC optimization

Since online SPE-HPLC method was mainly used to study drug-protein interaction, the experimental conditions should simulate the physiological environment in human body. 10 mM PBS (PH 7.4) was selected as the SPE cartridge mobile phase because its osmotic pressure and ion concentration were generally consistent with the human physiological environment. Moreover, the sample plate and the column compartment temperature were set at 37 °C, which also closed to the human body temperature. All the analytes had the very same SPE conditions close to the human conditions in order to guarantee the reliability of the results.

The online SPE procedure was optimized to realize high absolute recovery of test standards onto the SPE cartridge. Three kinds of commercial SPE cartridges including LiChrospher® RP-18 ADS, CAPCELL MF Ph-1 and Oasis® MAX were evaluated for the retention of all analytes. CAPCELL MF Ph-1 showed weak retention for phenylbutazon and warfarin sodium. Piroxicam could only be retained in Oasis® MAX that R_ab_ is 99.6%. LiChrospher® RP-18 ADS was chosen because of the better retention for 12 analytes except piroxicam, which R_ab_ was within the range of 96.1 ~ 102.6% (Table 2).

Before starting the online SPE-LC wizard, the matrix depletion time (*T*_m_), analyte break-through time (*T*_a_) and transfer time (*T*_t_) parameters must be determined in a separate experiment^13^. *T*_m_ was the time it took at a given flow rate of 1 mL/min to completely elute the sample matrix from the SPE cartridge. The SPE cartridge was directly coupled to the UV detector. After injection of 5 μL sample, the elution profile of the sample matrix was recorded (i.e. the UV detector set at a wavelength of 280 nm was appropriate to monitor the protein matrix) (Supplementary Fig. 2). Complete elution of the matrix was obtained (*T*_m_ = 0.5) when the detector signal reached the baseline again. Finally, *T*_m_ was set 1 min. *T*_a_ was the time it took at a flow rate of 1 mL/min until the target analytes started to elute from the SPE cartridge. The SPE cartridge was also directly coupled to the UV detector. After injection of a standard solution of the analytes, the elution profile was recorded. To ensure the complete extraction and recovery of the analytes, *T*_a_ should be greater than *T*_m_. *T*_t_ was the time it took at a flow rate of 1 mL/min to completely elute the analytes from the SPE cartridge and to transfer them to the analytical LC column. First, a standard solution of the analytes was injected onto the SPE column. Then the valve was switched and the mobile phase delivered by the left pump elutes the analytes from the SPE column to the detector. As obtained from this trial, the optimal *T*_t_ was different according to different analytes.

### The recovery measurements

The detailed process of samples measurement involved four steps. (a) The conventional HPLC system (only analytical column without SPE cartridge) was used to analyze 10 μg/mL test standards with the same analytical column conditions (Table 4) in online SPE-HPLC system. A peak area of the standards was defined as A_1_. (b) The online SPE-HPLC system was used to analyze 10 μg/mL test standards. A peak area of the standards was defined as A_2_. (c) The online SPE-HPLC system was used to analyze 10 μg/mL plasma samples. A peak area of the plasma samples was defined as A_3_. (d) The values of A_2_ divided by A_1_ was defined as absolute recovery (R_ab_). The values of A_3_ divided by A_2_ was defined as relative recovery (R_re_). The formula was as follows


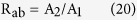



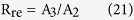


R_ab_ represented the absolute retention capability of SPE cartridge to test standards. R_re_ represented the relative retention capability of SPE cartridge to unbound drug in presence of plasma.

## Additional Information

**How to cite this article**: Li, P. *et al.* Characterization of plasma protein binding dissociation with online SPE-HPLC. *Sci. Rep.* **5**, 14866; doi: 10.1038/srep14866 (2015).

## Supplementary Material

Supplementary Information

## Figures and Tables

**Figure 1:**
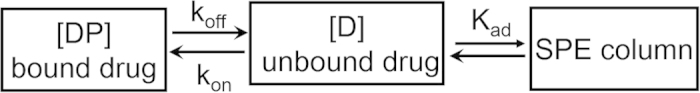
The interaction process in protein-drug-SPE column system.

**Table 1:** The PK parameters and physicochemical parameters of drugs.

Drugs	PPB rate(%)^a^^ ^14,15	T_1/2_ (h)^18^	Log P^25^	PKa^25^
Amitriptyline Hydrochloride	90.0	17–40	4.92	9.40
Dicoumarol	99.0	24–96	2.07	–
Estradiol	>95.0	36	4.01	10.33
Estriol	91.0	20	2.45	10.33
Fluoxetine Hydrochloride	94.0	2–4d	4.05	9.80
Norfluoxetine Hydrochloride	>90.0	7d	3.8	9.77
Phenylbutazon	97.8	30–175	3.16	4.50
Piroxicam	90.0	30–70	3.06	6.30
Warfarin Sodium	99.0	37–50	2.97	5.08
Diclofenac Sodium	99.5	1–2	4.51	4.15
Indomethacin	94.5	3–11	4.27	4.50
Nimodipine	95.0	1–2	3.05	5.40
Omeprazole	95.0	0.5–1	2.23	4.77

^a^PPB rate is a thermodynamic value which is measured the binding percentage in the steady state.

**Table 2:** The experimental data of 13 analytes.

Drugs	T_1_ (min)	T_2_ (min)	R_ab_ (%)	R_re_ (%)
Phenylbutazon	3.490	5.000	95.6	25.0
Warfarin Sodium	3.147	4.773	91.8	60.8
Dicoumarol	3.120	4.237	99.9	21.6
Estriol	5.317	6.177	102.6	70.2
Piroxicam	3.947	5.013	99.6	50.1
Amitriptyline Hydrochloride	9.410	9.657	101.0	94.6
Fluoxetine Hydrochloride	6.310	6.800	99.5	107.5
Norfluoxetine Hydrochloride	3.400	4.470	99.0	102.0
Estradiol	5.113	6.227	102.3	96.0
Nimodipine	4.087	5.277	94.1	90.5
Omeprazole	5.307	6.450	93.1	97.6
Diclofenac Sodium	4.947	6.017	100.8	96.3
Indomethacin	5.157	6.303	96.1	92.1

**Table 3:** The PK parameters and R_re_ values of drugs.

Drugs	T_1/2_ (h)^18^	V_d_ (L/kg)^25^	CL (mL/min/Kg)^25^	R_re_ (%)
Phenylbutazon	30–175	0.02–0.15	0.022	25.0
Warfarin Sodium	37–50	0.11 ± 0.01	0.045 ± 0.024	60.8
Dicoumarol	24–96	0.119	−12/−3	21.6
Estriol	20	–	–	70.2
Piroxicam	30–70	0.14	0.022	50.1
Amitriptyline Hydrochloride	17–40	8.3 ± 2.0	6.1 ± 1.7	94.6
Fluoxetine Hydrochloride	2–4d	20–45	–	107.5
Norfluoxetine Hydrochloride	7d	–	–	102.0
Estradiol	36	1	–	96.0
Nimodipine	1–2	0.9–2.3	–	90.5
Omeprazole	0.5–1	–	500–600 mL/min	97.6
Diclofenac Sodium	1–2	1.3	Oral CL = 622 mL/min; Renal CL < 1 mL/min	96.3
Indomethacin	3–11	0.3–1.6	0.11	92.1

**Table 4:** Analytical column conditions and SPE cartridge conditions.

Drugs	UV spectra (nm)	SPE cartridge	Analytical column	
			Acetonitrile (%)	10 mM PBS (%)
Amitriptyline Hydrochloride	239	LiChrospher RP-18 ADS	70	30
Diclofenac Sodium	283		35	65
Dicoumarol	277		45	55
Estradiol	281		50	50
Estriol	230		30	70
Fluoxetine Hydrochloride	228		55	45
Indomethacin	267		35	65
Nimodipine	237		70	30
Norfluoxetine Hydrochloride	228		60	40
Omeprazole	303		35	65
Phenylbutazon	267		35	65
Warfarin Sodium	310		30	70
Piroxicam	357	Waters Oasis MAX	55	45
