# Supplementary material for: Characterization of plasma protein binding dissociation with online SPE-HPLC
Source: Sci Rep. 2015 Oct 13;5:14866. doi: 10.1038/srep14866 (PMC4602217; doi:10.1038/srep14866)
Supplement: Supplementary Information [file srep14866-s1.pdf]

Characterization of plasma protein binding dissociation with online SPE-HPLC

Ping Li[1,2,3], Yiran Fan[1,2,3], Yunlong Wang[4], Yaxin Lu[1,2, &*], Zheng Yin[1,2, &*]

Supplementary Information

Selection of 13 drugs:

An analysis of the fractions bound to plasma protein of therapeutic drugs (more than 500) on the market indicated an even distribution with 43% of the drugs showing values in excess of 90%[1]. Meanwhile, more than 600 kinds of drugs reported in the literature were analyzed and found that the drugs with $T_{1/2}$ >8h accounted for about 35%, and the drugs with $T_{1/2}$>24h accounted for 18%[2]. According to the above data, it can be speculated the drugs with the percentage of protein binding greater than 90% and $T_{1/2}$>24h accounted for about 7.7% in small molecule drug library. It can be estimated that there are around 1500 frequently described drugs on the market today[1] and the drugs with $T_{1/2}$>24h with greater than 90% PPB are just 116 kinds.

We had chosen 13 kinds of drugs with high PPB (>90% PPB) [1, 3] that could be further divided into two categories: one with short half-life ($T_{1/2}$<4h), and the other with long half-life ($T_{1/2}$>24h) [2] (**Fig.1**).

The elution profile of plasma protein (**Fig.2**):

The UV detector was set at a wavelength of 280 nm to monitor the protein. After injection of 5 μL sample, the plasma protein was flushed out quickly in 0.1-0.5 min at flow rate of 1 mL/min.

References

1. Kratochwil, N.A., Huber, W., Muller, F., Kansy, M. & Gerber, P.R. Predicting plasma protein binding of drugs: a new approach. *Biochem. Pharmacol.* 64, 1355-1374(2002).
2. Regenthal, R., Krueger, M., Koeppel, C. & Preiss, R. Drug levels: Therapeutic and toxic serum/plasma concentrations of common drugs. *J. Clin. Monit. Comput.* 15, 529-544 (1999).
3. Zhang, F., Xue, J., Shao, J. & Jia, L. Compilation of 222 drugs' plasma protein binding data and guidance for study designs. *Drug Discov. Today* 17, 475-485 (2012).

Table Legends

Table 1 The influence of PPB and binding kinetics on ADME properties in vivo.

Table 1 The influence of PPB and binding kinetics on ADME properties in vivo

**PPB**	**Drug-protein dissociation**	**ADME properties**
High	Slow	Limitation
High	Fast	Permission
Low	Slow	Permission
Low	Fast	Permission

Figure Legends

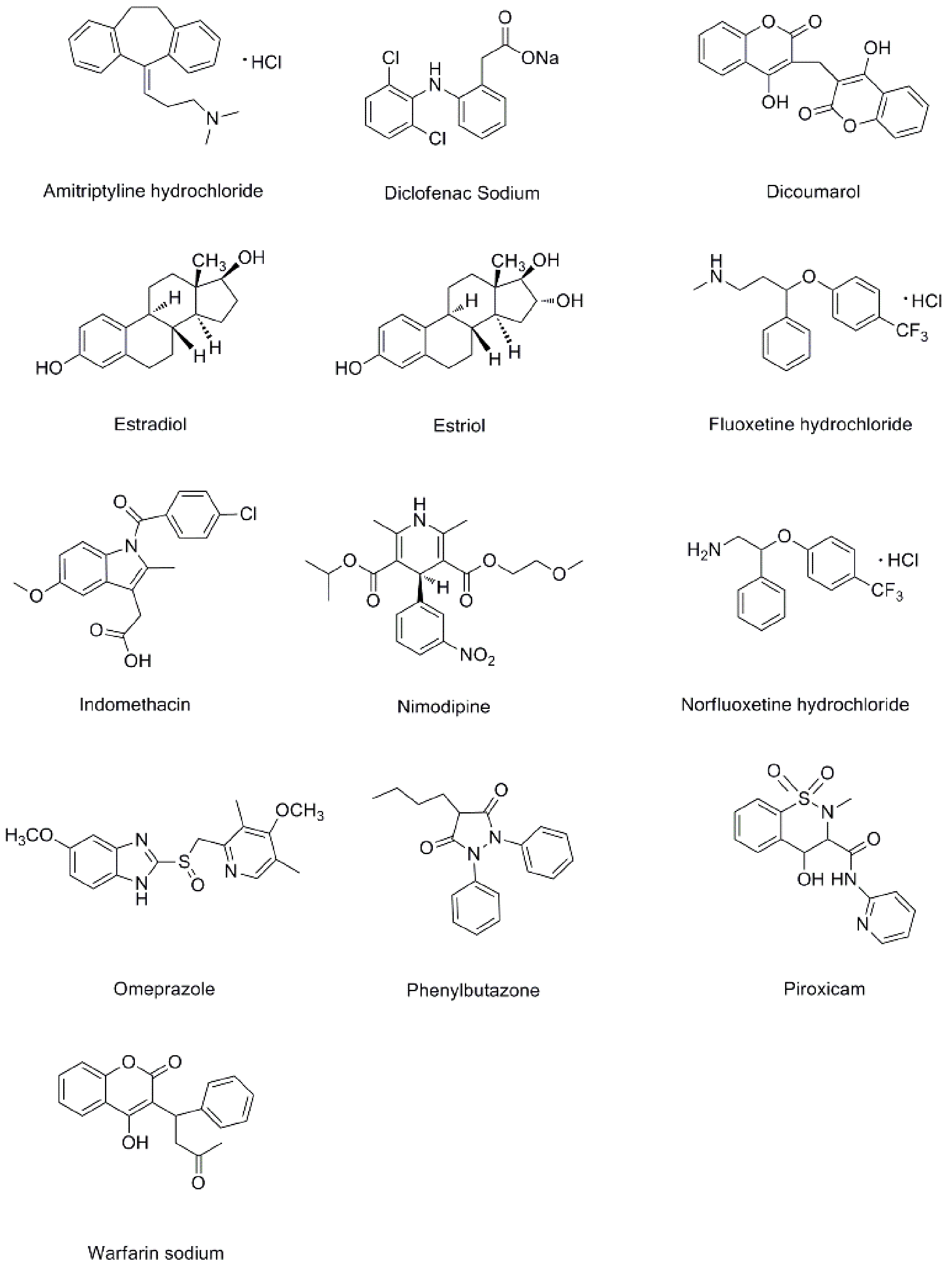

Figure 1 Chemical structures of the drugs.

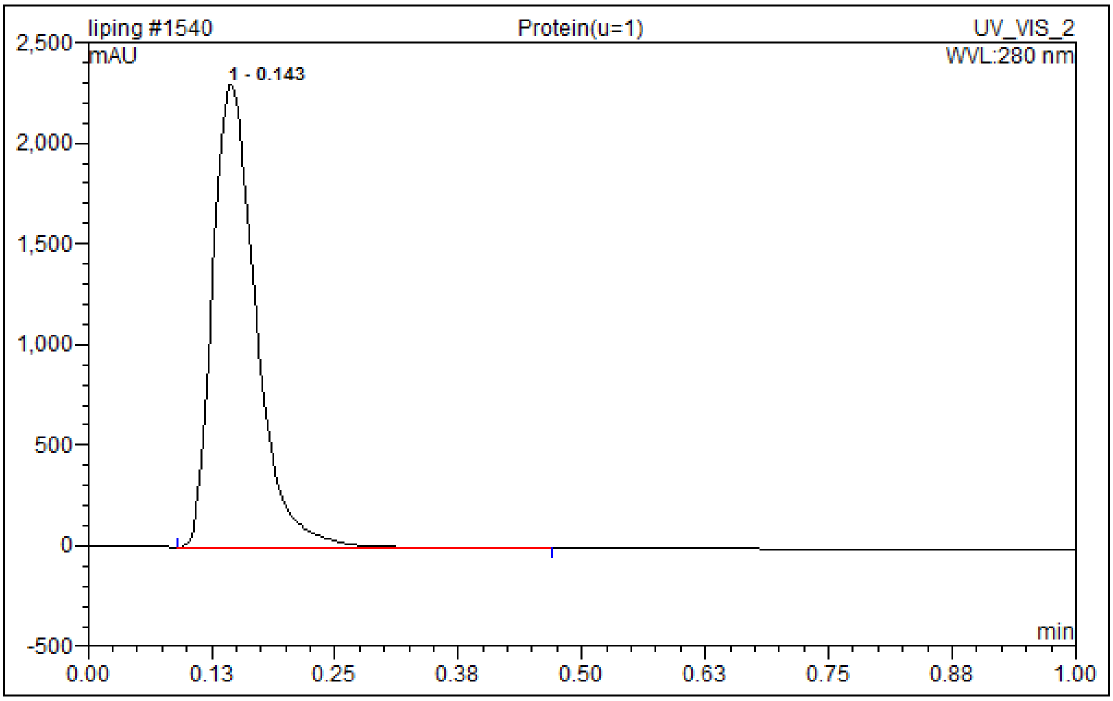

Figure 2 The elution profile of plasma protein.
